# Classification and staging of retinoblastoma

**Published:** 2018-06-03

**Authors:** Ido Didi Fabian, Ashwin Reddy, Mandeep S Sagoo

**Affiliations:** 1 Consultant Ocular Oncologist: Ocular Oncology Service, Goldschleger Eye Institute, Sheba Medical Center, Tel Aviv, Israel.; 2 Lead Clinician for Ophthalmology and Retinoblastoma Services: Royal London Hospital, London, UK.; 3 Retinoblastoma Service: Royal London Hospital; Ocular Oncology Service NIHR Biomedical Research Centre for Ophthalmology, Moorfields Eye Hospital and UCL Institute of Ophthalmology, London, UK.

**Classifying and staging retinoblastoma is an essential first step when planning how to manage a child with the condition; it also gives important information about prognosis.**

**Figure:**
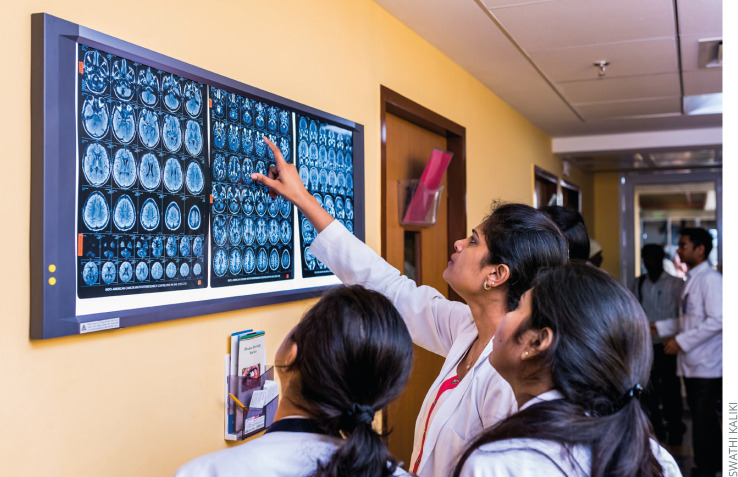
Doctors look for signs that retinoblastoma has spread via the optic nerve. INDIA

Classification schemes in cancer are mainly used to compare the results of different treatments and to enable a prognosis to be given.

## Classification of extraocular disease

If retinoblastoma is left untreated, it will extend beyond the eye. Unfortunately, this is the type most commonly seen in low- and middle-income countries. The tumour can penetrate the globe wall and be visible in and around the eye. It can also reach the central nervous system via the optic nerve, or it can spread to other parts of the body via the blood stream (metastases).

In 2006, Chantada and colleagues developed the International Retinoblastoma Staging System (IRSS; Table 1).^1^ It sub-classifies the disease from stage 0-IV. Stage 0 is intraocular disease, usually having a good outcome with treatment, and stage IV is retinoblastoma with metastases, which has a poor prognosis.

## Classification of intraocular disease

For intraocular retinoblastoma, the first classification system was introduced by Reese and Ellsworth (R-E) in the 1960s to predict the chances of saving the eye following external beam radiotherapy. When intravenous chemotherapy for intraocular retinoblastoma was introduced in the 1990s, the R-E classification system was no longer appropriate and a new classification scheme, the International Intraocular Retinoblastoma Classification (IIRC) scheme, was developed.^2^

The IIRC scheme groups tumours from A-E, depending on their size, location, and additional features, including the presence of retinoblastoma ‘seeds’ (small colonies of cancerous cells in the vitreous) and/or retinal detachment.

**Table 1:** International Retinoblastoma Staging System (IRSS)

Stage	Clinical Description
**0**	Patient treated conservatively
**I**	Eye enucleated, completely resected histologically
**II**	Eye enucleated, microscopic residual tumour
**III**	Regional extension
**a.**	Overt orbital disease
**b.**	Preauricular or cervical lymph node extension
**IV**	Metastatic disease
**a.**	Heamatogenous metastasis (without central nervous system involvement) 1 Single lesion 2 Multiple lesions
**b.**	Central nervous system extension (with or without any other site of regional or metastatic disease) 1 Prechiasmatic lesion 2 Central nervous system mass 3 Leptomeningeal and cerebrospinal fluid disease

**Figure 1:**
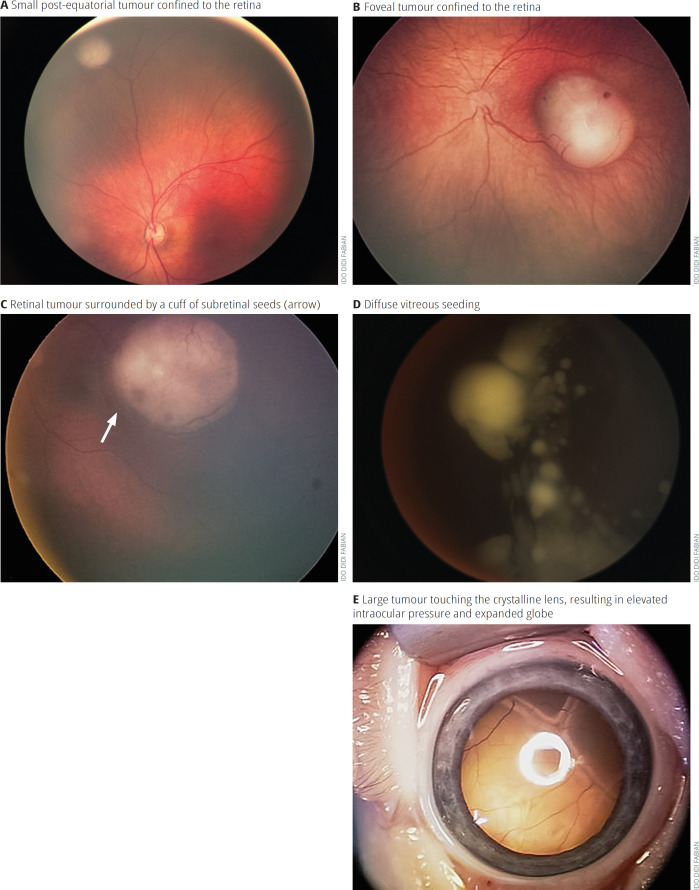
Intraocular retinoblastoma: stages A–E

The cTNMH scheme is available in the online edition of this article. www.cehjournal.org

Shields and colleagues developed a modified scheme, the Intraocular Classification of Retinoblastoma (ICRB), which differed from the IIRC mainly in the definitions of the advanced groups, D and E. In 2006, the ICRB scheme was found to successfully predict the outcome of intravenous chemotherapy.^3^

- For eyes in groups A-C: the globe could be salvaged in ≥90% of eyes.
- For eyes in group D: the globe could be salvaged in 47% of eyes.
- Group E eyes underwent primary enucleation and were excluded from analysis.

Both the IIRC and ICRB classification systems (see Table 2, overleaf) are now used as the main classification schemes for intraocular retinoblastoma and serve clinicians and researchers across the world.^4^ The images in Figure 1 correspond to each category.

Intravenous chemotherapy was found to be effective in treating tumours confined to the retina; however, vitreous seeds seemed to be resistant to the treatment. In 2012, Munier and colleagues developed a technique for injecting chemotherapy directly into the vitreous cavity.^5^ They used a classification system that grouped retinoblastoma seeds, based on their morphology and size, as dust, spheres and clouds. Since the introduction of the intravitreal technique, the classification of vitreous seeds is now commonly used to predict the number of injections needed to control the various seed types.^6^

Another classification system that is used for all cancer types, including retinoblastoma, was created by the American Joint Committee on Cancer (AJCC). The TNM scheme classifies cancer according to involvement of the primary site: tumour (T), lymph nodes (N) and presence of systemic metastasis (M). The recently published 8th edition includes a hereditary (H) component for Rb, making it the cTNMH scheme (c for clinical). The cTNMH categories are based on whether the tumour burden is determined to be intraretinal, intraocular, advanced intraocular or extraocular.^7^ The TNM scheme also has a pathological (pTNM) sub-classification which is widely used by ophthalmic pathologists. Read this article online to see the full scheme: **www.cehjournal.org**.

## Summary

Classification and staging systems for retinoblastoma have evolved as new treatments became available. Several schemes are currently available. For disease confined to the globe, the IIRC, ICRB and cTNMH systems are available, along with additional classifications describing vitreous seeds. For extraocular disease, the IRSS and cTNMH schemes can be used.

**Table 2:** Classification systems for Intraocular Retinoblastoma

	International Intraocular Retinoblastoma Classification (IIRC)	Intraocular Classification of Retinoblastoma (ICRB)
**Group A (very low risk)**	All tumours are 3 mm or smaller, confined to the retina and at least 3 mm from the foveola and 1.5 mm from the optic nerve. No vitreous or subretinal seeding is allowed	Retinoblastoma ≤ 3 mm (in basal dimension or thickness)
**Group B (low risk)**	Eyes with no vitreous or subretinal seeding and discrete retinal tumour of any size or location. Retinal tumours may be of any size or location not in group A. Small cuff of subretinal fluid extending ≤5 mm from the base of the tumour is allowed	Retinoblastoma > 3 mm (in basal dimension or thickness) or • Macular location (≤3 mm to foveola) • Juxtapapillary location (≤1.5 mm to disc) • Additional subretinal fluid (≤3 mm from margin)
**Group C (moderate risk)**	Eyes with focal vitreous or subretinal seeding and discrete retinal tumours of any size and location. Any seeding must be local, fine, and limited so as to be theoretically treatable with a radioactive plaque. Up to one quadrant of subretinal fluid may be present	Retinoblastoma with: • Subretinal seeds ≤ 3 mm from tumour • Vitreous seeds ≤ 3 mm from tumour • Both subretinal and vitreous seeds ≤ 3 mm from tumour
**Group D (high risk)**	Eyes with diffuse vitreous or subretinal seeding and/or massive, non-discrete endophytic or exophytic disease Eyes with more extensive seeding than Group C Massive and/or diffuse intraocular disseminated disease including exophytic disease and >1 quadrant of retinal detachment. May consist of ‘greasy’ vitreous seeding or avascular masses. Subretinal seeding may be plaque-like	Retinoblastoma with: • Subretinal seeds > 3 mm from tumour • Vitreous seeds > 3 mm from tumour • Both subretinal and vitreous seeds > 3 mm from retinoblastoma
**Group E (very high risk)**	Eyes that have been destroyed anatomically or functionally with one or more of the following: Irreversible neovascular glaucoma, massive intraocular haemorrhage, aseptic orbital cellulitis, tumour anterior to anterior vitreous face, tumour touching the lens, diffuse infiltrating retinoblastoma and phthisis or pre-phthisis	• Extensive retinoblastoma occupying >50% globe or with • Neovascular glaucoma • Opaque media from haemorrhage in anterior chamber, vitreous or subretinal space • Invasion of postlaminar optic nerve, • choroid (>2 mm), sclera, orbit, anterior chamber
